# Suppressive Effects of the *Gynura bicolor* Ether Extract on Endothelial Permeability and Leukocyte Transmigration in Human Endothelial Cells Induced by TNF-*α*

**DOI:** 10.1155/2020/9413724

**Published:** 2020-12-22

**Authors:** Shu-Ling Hsieh, Jyh-Jye Wang, Kuan-Hua Su, Ying-Lan Kuo, Shuchen Hsieh, Chih-Chung Wu

**Affiliations:** ^1^Department of Seafood Science, National Kaohsiung University of Science and Technology, Kaohsiung 81157, Taiwan; ^2^Department of Nutrition and Health Science, Fooyin University, Kaohsiung 83102, Taiwan; ^3^Graduate Institute of Medical Sciences, Chang Jung Christian University, Tainan 71101, Taiwan; ^4^Department of Chemistry, National Sun Yat-Sen University, Kaohsiung 80424, Taiwan; ^5^Department of Food and Nutrition, Providence University, Taichung 43301, Taiwan

## Abstract

*Gynura bicolor* (Roxb. and Willd.) DC (*G. bicolor*) is generally used as a dietary vegetable and traditional herb in Taiwan and the Far East. *G. bicolor* exerts antioxidant and anti-inflammatory effects and regulates blood lipids and cholesterol. However, the effects of *G. bicolor* on endothelial transmigration and atherosclerosis are not clear. The present study investigated the effects of *G. bicolor* on endothelial permeability and transmigration in human endothelial cells. We prepared *G. bicolor* ether extract (GBEE) for use as the experimental material. Under TNF-*α* stimulation, HL-60 cell adherence to EA.hy926 cells, the shape of EA.hy926 cells, and the expression of adhesion molecules and transmigration-related regulatory molecules were analysed after pretreatment with GBEE for 24 h. GBEE inhibited leukocyte adhesion to endothelial cells, reduced intercellular adhesion molecule-1 (ICAM-1) and platelet endothelial cell adhesion molecule-1 (PECAM-1) expressions, and decreased endothelial monolayer permeability. GBEE also reduced paracellular transmigration by reducing the levels of reactive oxygen species (ROS), Src phosphorylation, and vascular endothelial-cadherin (VE-cadherin) phosphorylation. GBEE reduced transcellular migration via inhibition of Ras homolog family member A (RhoA) and Rho-associated protein kinase (ROCK) expression and phosphorylation of the ezrin-radixin-moesin (ERM) protein. Incubation of EA.hy926 cells with GBEE for 8 h and stimulation with TNF-*α* for 3 h reduced the phosphorylation of the inhibitor of kappa B (I*ĸ*B) and DNA-binding activity of nuclear factor-*ĸ*B (NF-*ĸ*B). These results suggest that GBEE has a protective effect against endothelial dysfunction via suppression of leukocyte-endothelium adhesion and transmigration.

## 1. Introduction

*Gynura bicolor* (Roxb. and Willd.) DC (*G. bicolor*) is widespread in South Asia and the Far East, and it is generally used as a dietary vegetable and traditional herb in Taiwan. Previous studies showed that *G. bicolor* exhibited neuroprotective [1], liver-protective [2], hypoglycaemic [3], antioxidant [4], and anticancer properties [5] and promoted iron bioavailability [6]. Our previous study found that *G. bicolor* had anti-inflammatory [7] and antioxidant [8] effects and decreased serum total cholesterol, serum total triacylglycerol levels [8], and regulated immune response [9]. However, beyond its role in the regulation of blood lipids, the modification of lipoprotein levels [8] and raise antioxidative enzyme activity [9] during cardiovascular disease formation, the effects of *G. bicolor* on endothelial permeability, and leukocyte transmigration in atherosclerosis formation are not clear.

Atherosclerosis is one of the major causes of mortality worldwide, and vascular endothelial system dysfunction is an important cause of atherosclerosis [10]. Chronic pathological stimuli, such as diabetes, dyslipidaemia, inflammation, and oxidative stress, initiate endothelial dysfunction and vascular dysfunction, which lead to the development of atherosclerotic arterial disease [11]. Transmigration is the process by which leukocytes roll on the vascular endothelium, adhere to the endothelium, and invade across the endothelial cell-cell monolayer, and it occurs in the early stage of atherosclerosis formation [12]. The expression of intercellular adhesion molecule-1 (ICAM-1), vascular cell adhesion protein-1 (VCAM-1), and platelet endothelial cell adhesion molecule-1 (PECAM-1) plays an important role in transmigration. Activation of these adhesion molecules triggers the transformation of endothelial cells, changes the permeability of the endothelial cell-cell monolayer, and induces transmigration across the endothelial monolayer [13]. The transmigration of leukocytes consists of paracellular and transcellular transmigration activation [14], which are activated by oxidative stress, inflammation, and adhesion molecule activation [15]. Endothelial cell-cell junctions play an important role in transmigration. Therefore, maintaining endothelial cell-cell monolayer integrity and reducing transmigration are beneficial and have cardiovascular protective effects against atherosclerosis.

Nuclear factor-*ĸ*B (NF-*ĸ*B) is a crucial transcription factor that is involved in various physiological and pathological effects [16]. Activated NF-*ĸ*B binds to the cis-acting *ĸ*B enhancer element of target genes, such as ICAM1, E-selectin, Rho-associated protein kinase (ROCK), and Ras homolog family member A (RhoA), and activates their transcription [17]. Whether GBEE regulates these adhesion and transmigration molecules is an important issue.

The present study investigated *G. bicolor* ether extract (GBEE) mediated regulation of endothelial permeability and leukocyte transmigration in human endothelial cells. To determine the effect of GBEE on adhesion ability and endothelial cell-cell monolayer permeability, leukocyte adherence to endothelial cells, endothelial cell shape, the expression of adhesion molecules, phosphorylation of VE-cadherin, and the permeability of the endothelial cell-cell monolayer were analysed. To investigate the mechanism by which GBEE regulates the paracellular and transcellular transmigration pathways, the levels of transmigration-related regulatory molecules were measured. NF-*ĸ*B signalling was analysed to determine the regulatory effect of GBEE on transmigration and whether it occurred via the modification of NF-*ĸ*B signalling.

## 2. Materials and Methods

### 2.1. Preparation of GBEE

Fresh *G. bicolor* was purchased from the village of Yuanshan (Ilan, Taiwan) and identified by Yen Hsueh Tseng, Ph.D., based on a voucher specimen growing in the Department of Forestry, National Chung Hsieh University (NCHU, Taichung, Taiwan). A voucher specimen (TCF13549) of the newly purchased *G. bicolor* was deposited at NCHU.

To prepare GBEE, the fresh leaves of *G. bicolor* were collected, cleaned, and blended in cold water (4°C, w/w : 1/1). The homogenates were extracted with ether (v/v : 1/1) on a stir plate for 6 h at 4°C. The extract was centrifuged at 250 × g at 4°C for 10 min. The supernatant was filtered and concentrated using a rotary vacuum dryer (EYELA-Tokyo Rikakikai Co., Ltd., Tokyo, Japan) (45°C), and the concentrated product was dried in a freeze dryer at −43°C. The percentage yield of the GBEE was 0.3% (w/w).

### 2.2. Cell Culture and GBEE Treatment

EA.hy926 cells were established via fusion with primary human umbilical vein cells and used in this study as a model of the vascular endothelium to investigate the effects of GBEE on adhesion and transmigration in the vascular endothelial monolayer of the circulatory system. HL-60 human leukaemia cells derived from peripheral blood leukocytes and obtained using leukapheresis are widely used to study interactions of adhesion and adhesion molecules [18]. EA.hy926 and HL-60 cells were purchased from the Bioresource Collection and Research Center (BCRC, Hsinchu, Taiwan). EA.hy926 cells (at passages 40–62) and HL-60 cells (at passages 23–46) were maintained in DMEM supplemented with 10% foetal bovine serum and 1% penicillin/streptomycin at 37°C in a 5% CO_2_ humidified atmosphere.

EA.hy926 cells were plated at a density of 1 × 10^4^ per 30 mm culture dish and incubated until reaching 90% confluence. To determine the effects of GBEE on cell viability, adhesion, and transmigration, the cultured cells were pretreated with 10, 50, or 100 *μ*g/mL GBEE for 8 h and then stimulated with or without 10 ng/mL TNF-*α* (R&D Systems, Inc., Minneapolis, MN, USA) for 3 h. TNF-*α* was used in this study to induce oxidative stress and endothelial dysfunction [19], and the GBAE treatment time and the dose were confirmed in a series of pretests of adhesion ability and the phosphorylation of VE-cadherin, two adhesion ability, and permeability indicator, in this present study. The present study dissolved the GBEE in dimethyl sulfoxide (DMSO, Sigma-Aldrich Co., St. Louis, MO. USA), and a group treated with only DMSO was used as a control group. Groups treated with only 10 ng/mL TNF-*α* for the last 3 h or 100 *μ*g/mL GBEE for the first 8 h were used as control groups.

### 2.3. Cell Viability Analysis

Based on the results of our preliminary test to determine the experimental concentrations of GBEE, EA.hy926 cells were treated with 10, 50, or 100 *μ*g/mL GBEE for 24 h for the cell viability assay. EA.hy926 cells were pretreated with 10, 50, or 100 *μ*g/mL GBEE for 8 h and then stimulated with or without 10 ng/mL TNF-*α* for 3 h for the cell viability assay. Cell viability was evaluated using the 3-(4, 5-dimethyl-2-yl)-2, 5-diphenyl tetrazolium bromide (MTT, Sigma-Aldrich Co.) reduction assay, and morphological examination was performed as described by Denizot and Lang [20]. The cells were incubated with DMEM containing MTT reagent (5 *μ*g/mL) at 37°C for 3 h. The medium was removed, and the cells were washed twice with phosphate-buffered saline (PBS, 3.2 mM Na_2_HPO_4_, 0.5 mM KH_2_PO_4_, 1.3 mM KCl, 135 mM NaCl, and pH 7.4). Formazan formation, as an indicator of cell viability, was solubilized via the addition of 1 mL of acidified isopropanol into each plate. After 15 min of extraction, the extent of formazan production was determined by reading the absorbance at 570 nm using an enzyme-linked immunosorbent assay (ELISA) reader (BioTek Instruments Inc., Winooski, VT, USA). A phase-contrast inverted fluorescence microscope was used to determine morphological changes (Olympus IX51, Olympus, Tokyo, Japan).

### 2.4. Adhesion Assay

An adhesion assay was performed as described by Braut-Boucher et al. [21] with modifications. EA.hy926 cells were pretreated with 10, 50, or 100 *μ*g/mL GBEE for 8 h and then stimulated with or without 10 ng/mL TNF-*α* for 3 h. The EA.hy926 cells were washed in PBS and cocultured for 1 h with HL-60 cells labelled with 10 *µ*M 2′, 7′-bis-(2-carboxyethyl)-5-(and-6)-carboxyfluorescein (BCECF, Thermo Fisher Scientific, Carlsbad, CA, USA) for 1 h. After washing in PBS, the morphology of BCECF-stained HL-60 cells was measured under an inverted fluorescence microscope (Olympus IX51, Olympus, Tokyo, Japan). The BCECF-stained cells were collected and measured fluorometrically using an ELISA reader (BMG Labtech GmbH, Offenburg, Germany) with excitation and emission wavelengths of 500 and 530 nm, respectively.

### 2.5. Atomic Force Microscopy (AFM) Examination

EA.hy926 cells (5 × 10^4^ cells/30°mm plate) were seeded on cover slides in 6-well culture plates overnight and treated with 10, 50, or 100 *μ*g/mL GBEE for 8 h, followed by stimulation with or without 10 ng/mL TNF-*α* for 3 h. For AFM imaging, the attached cultured cells on slides were removed, washed three times with PBS, fixed with 4% paraformaldehyde for 20 min, and washed three times with PBS, which was exchanged with ultrapure water.

An atomic force microscope (MFP-3D, Asylum Research, Santa Barbara, CA, USA) was used to characterize the cells under ambient conditions [22]. A silicon cantilever (NanoWorld, Switzerland, Arrow FMR) with a measured spring constant of 2.8 N/m was used to image the melanoma cells. Images were collected in air using the contact mode at a scan rate of 1 Hz.

### 2.6. Measurement of Transepithelial Resistance (TEER)

TEER is a quantitative measurement of the barrier integrity of a monolayer used to examine cell-cell integrity and permeability [23]. To measure the TEER, EA.hy926 cell monolayers (5 × 10^4^ cells/well) were cultured on semipermeable filter (0.22 *μ*m) inserts that served as a partition to the apical and basolateral compartments. EA.hy926 cells (5 × 10^4^ cells/30 mm plate) were seeded on cover slides in 6-well culture plates overnight, then treated with 10, 50, or 100 *μ*g/mL GBEE for 8 h, followed by stimulation with or without 10 ng/mL TNF-*α* for 3 h. After the above treatments, one electrode was placed in the upper compartment, and another electrode was placed in the lower compartment, with the electrodes separated by the cellular monolayer. The measurement procedure included the measurement of the resistance of the semipermeable membrane as a blank. The cell-specific resistance was measured in units of Ω. TEER values were obtained by subtracting the TEER measured at a groove in the cell culture dish from the measurement in the presence of a cell layer. These measurements were acquired using a Millicell-ERS voltohmmeter (Millipore Continental Water Systems, Bedford, MA, USA).

### 2.7. Analysis of the Expression of Regulatory Proteins Involved in Transmigration

EA.hy926 cells (5 × 10^5^/30 mm plate) were used to analyse the expression of adhesion molecules (ICAM-1 and PECAM-1), adhesion junction proteins (phosphorylation-vascular endothelial cadherin (p-cadherin) and VE-cadherin), adhesion junction regulatory proteins (p-Src, Src, Ras homolog family member A (RhoA), Rho-associated protein kinase (ROCK), and p-ezrin-radixin-moesin (ERM)),, and NF-*ĸ*B signalling molecules (p-I*ĸ*B, I*ĸ*B, cytosolic NF-*ĸ*B, and nuclear NF-*ĸ*B (p65)).

EA.hy926 cells were pretreated with 10, 50, or 100 *μ*g/mL GBEE for 8 h and stimulated with or without 10 ng/mL TNF-*α* for 3 h. The cells were washed twice with cold PBS and harvested using 200 *μ*L of lysis buffer containing 10 mM Tris-HCl, 5 mM EDTA, 0.2 mM phenylmethylsulphonyl fluoride (PMSF) (Sigma-Aldrich Co.), and 20 *μ*g/ml aprotinin at pH 7.4. The cellular proteins were quantified following the method described by Lowry et al. [24].

Cellular protein (10 to 20 *µ*g) from each sample was added to 10% sodium dodecyl sulphate (SDS) polyacrylamide gels [25]. After electrophoresis, the proteins were transferred to polyvinylidene difluoride membranes [26], which were incubated with antibodies against ICAM-1, PCAM-1, p-VE-cadherin, VE-cadherin, p-Src, Src, RhoA, ROCK, p-PERM, ERM, p-I*ĸ*B, I*ĸ*B, NF-*ĸ*B, and nuclear NF-*ĸ*B (p65) at 37°C for 1 h and subsequently incubated with peroxidase-conjugated secondary antibodies. Bands were visualized using hydrogen peroxide/tetrahydrochloride diaminobenzidine or an enhanced chemiluminescence detection kit (Amersham Life Science, Buckinghamshire, UK) and quantified using a ChemiDoc MP imaging system (Bio-Rad Laboratories Inc., Hercules, CA, USA).

### 2.8. Statistical Analysis

The data were analysed using SPSS statistical analysis software for Windows, version 20.0 (IBM, Armonk, NY, USA). One-way analysis of variance and Duncan's multiple range tests were used to evaluate the significance of differences between mean values. ^abcd^Values were significantly different from the other groups. A *p* value less than 0.05 indicated a statistically significant difference.

## 3. Results

### 3.1. GBEE Did Not Reduce the Cell Viability of TNF-*α*-Induced EA.hy926 Cells

**Figure 1:**
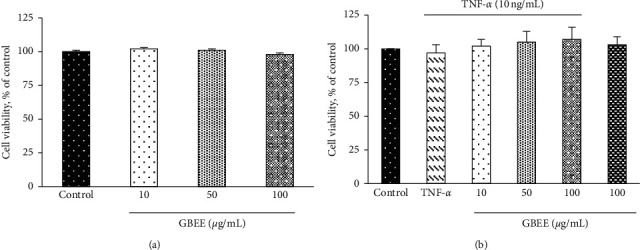
Effect of GBEE on cell viability in EA.hy926 cells. EA.hy926 cells (5 × 10^4^ cells/30 mm plate) were seeded and cultured overnight. (a) EA.hy926 cells treated with 10, 50, or 100 *μ*g/mL GBEE for 24 h. (b) EA.hy926 cells treated with 10, 50, or 100 *μ*g/mL GBEE for 8 h followed by stimulation with or without 10 ng/mL TNF-*α* for 3 h. Values were presented as means ± SDs (*n* = 3). ^abcd^Values not sharing the same letter were significantly different, as shown by Duncan's test (*p* < 0.05).

The viability of EA.hy926 cells treated with 10, 50, or 100 *μ*g/mL GBEE for 24 h was not significantly different from control cells (Figure 1(a)). The cell viability of EA.hy926 cells did not significantly differ between the groups treated with 10, 50, or 100 *μ*g/mL GBEE and TNF-*α* (approximately 102–107%), only TNF-*α* (97%), and only GBEE (101%) and the control group (100%) (Figure 1(b)). Morphological examination using inverted microscopy revealed no significant differences in the cell number and cell morphology between any GBEE group and the control group (data not shown). Therefore, treatment with 10, 50, or 100 *μ*g/mL GBEE did not affect the cell viability of EA.hy926 cells treated with TNF-*α*.

### 3.2. GBEE Reduced HL-60 Cell Adhesion to EA.hy926 Cells and TNF-*α*-Induced Changes in Cell Shape

**Figure 2:**
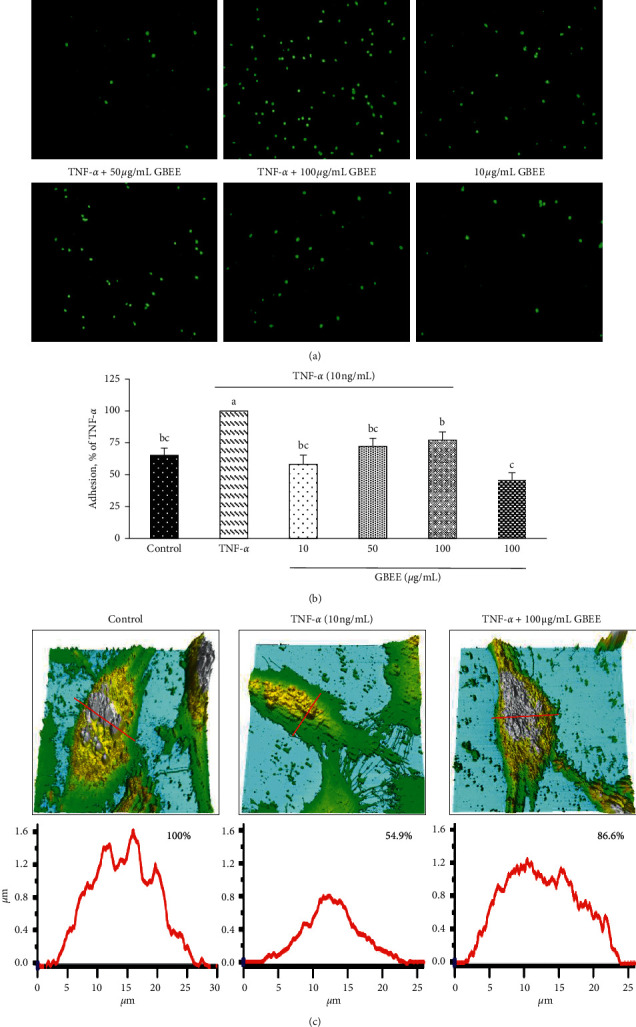
Effect of GBEE on HL-60 cell adhesion to EA.hy926 cells and analysis of TNF-*α*-induced changes in EA.hy926 cell height. EA.hy926 cells (5 × 10^4^ cells/30 mm plate) were seeded, cultured overnight, and treated with 10, 50, or 100 *μ*g/mL GBEE for 8 h followed by stimulation with or without 10 ng/mL TNF-*α* for 3 h. (a) HL-60 cells were prestained with 2′,7′-bis-(2-carboxyethyl)-5-(and-6)-carboxyfluorescein, and the adhesion of HL-60 cells to EA.hy926 cells was observed under a fluorescence microscope. (b) EA.hy926 cell staining was observed using a fluorescence reader with an excitation wavelength of 485 nm and an emission wavelength of 530 nm. (c) EA.hy926 cell height was observed using AFM. The height of the control cells was set at 100%. Values were presented as means ± SDs (*n* = 3). ^abc^Values not sharing the same letter were significantly different, as shown by Duncan's test (*p* < 0.05).

Fluorescence microscopy examination revealed that the GBEE treatment of TNF-*α*-induced EA.hy926 cells significantly reduced HL-60 cell adhesion to EA.hy926 cells (Figure 2). After EA.hy926 cells were incubated with 10, 50, or 100 *μ*g/mL GBEE for 8 h and stimulated with TNF-*α* for 3 h (Figures 2(a) and 2(b)), cell adhesion significantly decreased approximate 23–42% compared to the TNF-*α*-treated group (100%) (*p* < 0.05). These results demonstrate that GBEE decreased the ability of HL-60 cells to adhere to EA.hy926 cells and suggest that GBEE reduces leukocyte adhesion to an endothelial cell layer. Because 10, 50, or 100 *μ*g/mL GBEE reduced the adhesion ability, the 100 *μ*g/mL GBEE group was further used to investigate the mechanisms of cell height (morphological remodelling), cell junction protein, and gene regulation.

AFM technology was used to investigate the effects of GBEE on changes in EA.hy926 cell shape due to changes in cell-cell junctions. TNF-*α*-treated EA.hy926 cells, but not control cells, exhibited a flattened and partially collapsed shape (Figure 2(c)). The number of EA.hy926 cells stimulated with TNF-*α* and treated with 100 *μ*g/mL GBEE was higher (86.6% compared to the control) than the number of cells in the TNF-*α* group (54.9% compared to the control) and exhibited a less flattened shape (Figure 2(c)).

### 3.3. GBEE Inhibited Changes in the Adhesion Molecule and Junction Protein Expression and Permeability in TNF-*α*-Induced EA.hy926 Cells

**Figure 3:**
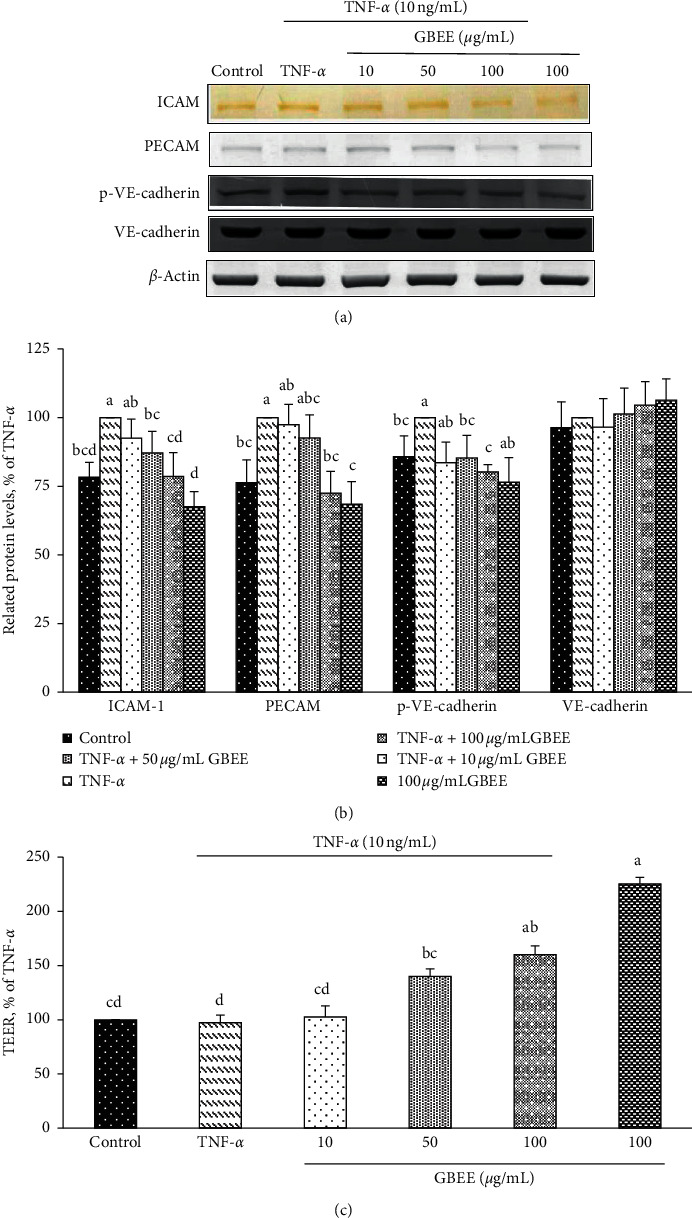
Expression levels of TNF-*α*-induced adhesion molecules and cell junction proteins. EA.hy926 cells (5 × 10^4^ cells/30 mm plate) were seeded, cultured overnight, and treated with 10, 50, or 100 *μ*g/mL GBEE for 8 h followed by stimulation with or without 10 ng/mL TNF-*α* for 3 h. (a) Immunoblot assays were performed to determine the expression levels of ICAM-1, PECAM-1, p-VE-cadherin, and VE-cadherin in EA.hy926 cells. (b) Protein expression in EA.hy926 cells was quantified using densitometry. The expression levels in the control group were set at 100%. (c) The effect of GBEE on transepithelial electrical resistance in EA.hy926 cells. Values were presented as means ± SDs (*n* = 3). ^abc^Values not sharing the same letter were significantly different, as shown by Duncan's test (*p* < 0.05).

To investigate the effects of GBEE on adhesion and adhesion junctions, changes in the expression of proteins related to cell adhesion and cell junctions in EA.hy926 were examined using immunoblotting. The expression levels of ICAM-1, PECAM-1, p-VE-cadherin, and VE-cadherin in EA.hy926 cells are presented in Figure 3(a). The treatment of EA.hy926 cells with 10, 50, or 100 *μ*g/mL GBEE and TNF-*α* significantly decreased approximate 49–76% and reduced PECAM-1 protein levels 27% under 100 *μ*g/mL GBEE treatment compared to the TNF-*α*-treated group (100%) (Figure 2(b)). After treatment with 10, 50, or 100 *μ*g/mL GBEE, the levels of p-VE-cadherin in EA.h926 cells were 83.6 ± 7.5, 85.3 ± 8.2, and 80.2 ± 2.7%, respectively, of the level in the TNF-*α*-treated group (*p* < 0.05) (Figure 2(b)). When EA.hy926 cells were treated with 10, 50, or 100 *μ*g/mL GBEE and TNF-*α*, no difference in the protein levels of VE-cadherin was observed compared with the control or TNF-*α*-treated groups (Figures 2(a) and 2(b)). These results indicate that GBEE suppressed the expression of this adhesion and junctional protein, and GBEE may exert an important antiadhesive effect and maintain cell junction function during TNF-*α* stimulation.

The effect of GBEE on the TEER of EA.hy926 cells was also examined. After treatment with 50 and 100 *μ*g/mL GBEE, the TEER of EA.hy926 cells was 141 ± 7% and 160 ± 8%, respectively (Figure 2(c)), and these values were significantly higher than those of the TNF-*α*-treated group (100%) (*p* < 0.05). These results demonstrated that GBEE decreased the gaps between cell-cell junctions and decreased permeability by reducing changes in the shape of EA.hy926 cells.

### 3.4. GBEE Regulated Paracellular and Transcellular Transmigration Regulatory Molecules in TNF-*α*-Induced EA.hy926 Cells

**Figure 4:**
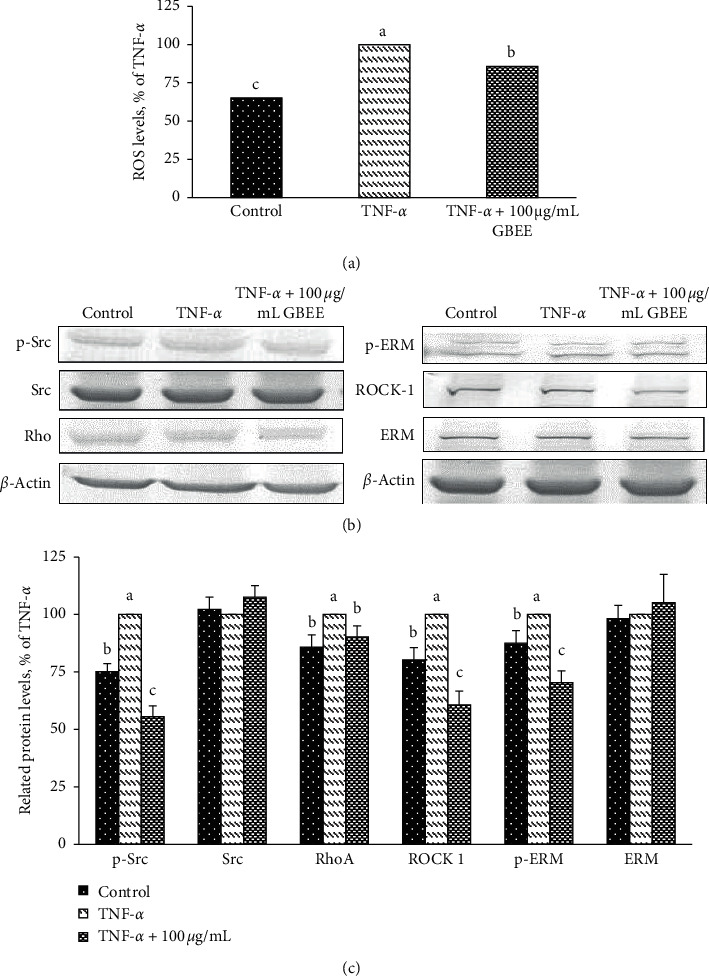
Effect of GBEE on transmigration regulatory molecules in EA.hy926 cells. EA.hy926 cells (5 × 10^4^ cells/30 mm plate) were seeded, cultured overnight, and treated with 100 *μ*g/mL GBEE for 8 h followed by stimulation with or without 10 ng/mL TNF-*α* for 3 h. (a) The effect of GBEE on ROS levels in EA.hy926 cells was determined. (b) The expression levels of p-Src, Src, RhoA, p-ERM, ROCK, and ERM in EA.hy926 cells were determined using the immunoblot assay. (c) The protein expression in EA.hy926 cells was quantified using densitometry; expression levels in the control group were set at 100%. Values were presented as means ± SDs (*n* = 3). ^abc^Values not sharing the same letter were significantly different, as shown by Duncan's test (*p* < 0.05).

The major molecules that regulate paracellular and transcellular transmigration were analysed in this study. As shown in Figure 4, the levels of reactive oxygen species (ROS) in EA.hy926 cells treated with 100 *μ*g/mL and TNF-*α* decreased significantly approximately 14% compared to the TNF-*α*-treated group (100%) (*p* < 0.05). p-Src and RhoA protein expressions decreased significantly, 44% and 10%, respectively, compared to the TNF-*α*-treated group (*p* < 0.05) (Figures 3(b) and 3(c)). However, Src protein levels were not altered in cells exposed to 100 *μ*g/mL GBEE and TNF-*α* (Figures 3(b) and 3(c)). However, when EA.hy926 cells were treated with 100 *μ*g/mL GBEE and TNF-*α*, ROCK and p-ERM expressions decreased significantly (60.7 ± 53 and 70.4 ± 12.2%, respectively) compared to the TNF-*α*-treated group (*p* < 0.05) (Figures 3(b) and 3(c)). These results showed that GBEE significantly reduced the levels of molecules that regulated paracellular and transcellular transmigration.

### 3.5. GBEE Reduced the Activation of NF-*ĸ*B Signalling in TNF-*α*-Induced EA.hy926 Cells

The results of immunoblot analysis showed that the phosphorylation of I*κ*B and nuclear NF-*κ*B levels decreased significantly, 24% and 16%, respectively, after 100 *μ*g/mL GBEE treatment (*p* < 0.05) (Figures 4(a) and 4(b)), but GBEE did not impact the protein levels of cytosolic I*κ*B and NF-*κ*B in EA.hy926 cells (Figures 4(a) and 4(b)). As shown in Figure 4(c), the DNA-binding activity of nuclear NF-*κ*B was significantly inhibited (49%) in cells treated with 100 *µ*g/mL GBEE.

## 4. Discussion

The present study showed that GBEE had a potential protective effect against atherosclerosis via suppression of leukocyte-endothelium transmigration. Our results showed that GBEE inhibited leukocyte adhesion to endothelial cells and reduced the adhesion molecule expression and endothelial monolayer permeability. GBEE reduced the phosphorylation of VE-cadherin by inhibiting ROS levels, the phosphorylation of Src, and the paracellular transmigration of TNF-*α*-induced EA.hy926 cells. GBEE also decreased RhoA and ROCK levels and the phosphorylation of ERM and inhibited the transcellular transmigration of TNF-*α*-induced EA.hy926 cells. These novel findings show that GBEE reduces paracellular and transcellular transmigration to maintain cell-cell monolayer integrity, reduce permeability, and prevent atherosclerosis.

Leukocytes roll and adhere to the endothelium of the vasculature in the early stage of atherosclerosis [27]. When these leukocytes roll on the vascular endothelium, endothelial adhesion molecules, such as ICAM-1, VCAM-1, and PECAM-1, are expressed and activated, which leads to adhesion between the endothelium and leukocytes [28]. Reduced adhesion molecule expression is one mechanism of decreased leukocyte adhesion to the vascular endothelium, which reduces plaque formation. GBEE significantly inhibited ICAM-1 and PECAM-1 expressions in the present study, which led to reduced HL-60 cell adhesion to EA.hy926 cells. These findings are similar to those of a previous study that showed that oligomeric proanthocyanidins from *Rhodiola rosea* (OPCRR) decreased the serum levels of TNF-*α*, IL-1*β*, IL-6, ICAM-1, and VCAM-1 and enhanced IL-10 levels in atherosclerotic rats, which improved endothelial dysfunction and atherosclerosis via decreased inflammation and the expression of adhesion molecules [29]. *Sorghum bicolor* L. Moench fermented with *Aspergillus oryzae* NK (fSBE) improved blood and vascular health by decreasing the levels of VCAM-1, ICAM-1, cyclooxygenase-2, and heme oxygenase-1 [30]. Our previous study showed that the *G. bicolor* ethanol extract decreased ICAM-1 and VCAM-1 expressions in TNF-*α*-induced EA.hy926 cells via potential antioxidant effects [9]. Inflammation and oxidative stress are the leading causes of atherosclerosis as a result of damage to the endothelium [11]. Oxidative stress activates redox signalling pathways, which leads to inflammatory insult [31]. An inflammatory state and the upregulation of inducible NOS (iNOS) expression lead to vascular remodelling. However, these structural changes may ultimately lead to vascular dysfunction [32, 33]. A previous study showed that GBEE reduced ROS, NO, and PGE_2_ levels and increased SOD activity in *in vitro* and *in vivo* models [7, 9]. The potential antioxidant and anti-inflammatory effects of GBEE may regulate adhesion molecules and reduce adhesion.

Before leukocyte invasion and transmigration across the endothelial monolayer, the shape of monolayer endothelial cells in the vasculature is transformed [34, 35]. The shrinking, retraction, and/or flattening of endothelial cells may lead to increased cell-cell monolayer permeability and the loss of integrity and trigger transmigration [36]. AFM examination indicated that EA.hy926 cells became flat and retracted after TNF-*α* induction in the present study. However, cotreatment with GBEE significantly reduced this flattening in TNF-*α*-induced EA.hy926 cells. As known, a normal endothelial shape and higher TEER indicate tight cell-cell junctions and the integrity of the cell-cell monolayer. TEER is a widely accepted quantitative indicator of the integrity and permeability of endothelial and epithelial monolayers in cell culture models [37]. Cell-cell junctions control endothelial cell-cell monolayer integrity and regulate the ability of ions, proteins, leukocytes, and macrophages to pass through this barrier [37]. The previous report showed some of the barrier models that have been widely characterized utilizing TEER include the blood-brain barrier (BBB), gastrointestinal (GI) tract, and pulmonary models [37]. Bachinger et al. showed that there are significantly higher TEER levels than the cell control group after intestinal porcine epithelial cells (IPEC-J2) were treated with *Angelica* root extracts for 24 h [38]. In the mouse vascular endothelial cell (mMVEC) culture model, puerarin, an active compound of *Pueraria lobata* (Willd.) Ohwi, also has significantly increased the TEER levels as compared to the cell control group [39]. A previous study showed that a dietary polyphenol compound, resveratrol, reduced endothelium monolayer permeability by increasing TEER and maintaining VE-cadherin levels to prevent atherosclerosis in patients with chronic kidney disease [40]. In the present study, when EA.hy926 cells were cotreated with GBEE and TNF-*α*, they had a higher TEER than the TNF-*α*-treated control group. The above studies show the raised TEER levels in the GI tract, vascular endothelium, and epithelium cells. All can enhance intestinal and vascular barrier integrin protective cell-cell monolayer dysfunction. The results of the present study showed that GBEE improved and maintained cell-cell monolayer integrity and reduced the increased permeability of TNF-*α*-induced EA.hy926 cells.

**Figure 5:**
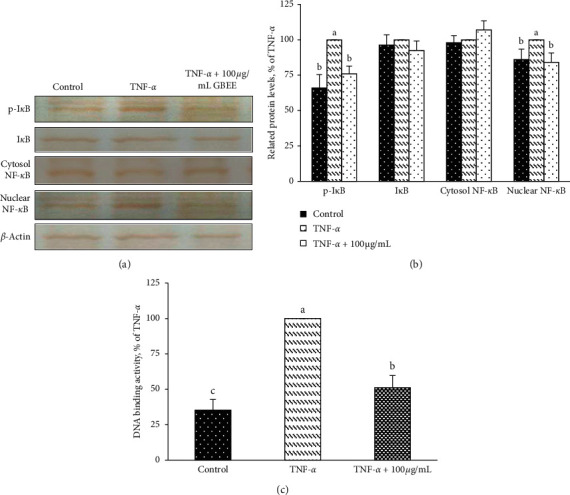
Effect of GBEE on NF-*κ*B signalling activation in EA.hy926 cells. EA.hy926 cells (5 × 10^4^ cells/30 mm plate) were seeded, cultured overnight, and treated with 100 *μ*g/mL GBEE for 8 h followed by stimulation with or without 10 ng/mL TNF-*α* for 3 h. (a) Immunoblot assays were performed to determine the expression levels of p-I*κ*B, I*κ*B, cytosolic NF-*κ*B, and nuclear NF-*κ*B in EA.hy926 cells. (b) Protein expression in EA.hy926 cells was quantified using densitometry; the expression levels in the control group were set at 100%. (c) The effect of GBEE on NF-*κ*B DNA-binding activity in EA.hy926 cells was determined. Values were presented as means ± SDs (*n* = 3). ^abc^Values not sharing the same letter were significantly different, as shown by Duncan's test (*p* < 0.05).

GBEE improved TNF-*α*-induced changes in the cell shape and the permeability of the endothelial cell-cell monolayer and regulated transmigration via regulating cell-cell junctions in the present study. Changes in the shape of endothelial cells lead to changes in endothelial cell-cell junctions. Endothelial cells primarily regulate cell-cell junctions and interactions via adhesion junctions and tight junctions [41]. Adhesion junctions join cells and have various cellular physiological effects, including the establishment and maintenance of cell-cell adhesions, actin cytoskeleton remodelling, intracellular signalling, and transcriptional regulation. Tight junctions regulate cell-cell monolayer permeability and maintain a specific barrier [42]. As shown in Figure 5, ICAM-1 expression and engagement increase the phosphorylation of Src, which causes the phosphorylation of VE-cadherin [12, 43]. The phosphorylation of VE-cadherin releases adhesion junctions and tight adhesion junctions to form an open channel, which leads to the movement of leukocytes, proteins, electrolytes, and solutes across the vascular monolayer. Figure 5 also shows that GBEE significantly reduced the phosphorylation of Src, VE-cadherin, and ERM, but it did not affect the levels of these proteins. These results showed that GBEE regulated Src, VE-cadherin, and ERM by reducing the phosphorylation of these proteins and confirmed that phosphorylation played an important role in the activation of Src, VE-cadherin, and ERM (12). ROS levels increase the phosphorylation of VE-cadherin and PECAM-1 expression. Therefore, increased ROS levels enhance the opening of cell-cell junctions by decreasing PECAM-1 levels and increasing VE-cadherin activation [44]. A previous study showed that luteolin suppressed adherens junction-associated monocyte paracellular transmigration by decreasing the PECAM-1 expression in TNF-*α*-induced THP-1 cells [45]. Mulberry polyphenol extract, which contains high levels of polyphenolic compounds, inhibited the expression of Src and reduced the expression of RhoA to affect F-actin cytoskeleton rearrangement, which inhibited A7r5 cell migration and atherosclerosis [46]. GBEE reduced the activation of the ICAM-1 expression and significantly decreased the levels of p-Src, ROS, PECAM-1, and p-VE-cadherin in TNF-*α*-induced EA.hy926 cells in the present study. These results showed that GBEE reduced paracellular transmigration. GBEE also regulated transcellular transmigration by reducing RhoA, ROCK, and p-ERM expressions (Figure 5). ICAM-1 activation triggers RhoA/ROCK signalling, and ICAM-1 induction increases RhoA levels to enhance the ROCK expression in endothelial cells. The subsequent phosphorylation of ERM increases and regulates cell remodelling and transcellular transmigration. RhoA/Rho kinase-mediated actin contractility may contribute to vascular function as a mechanosensory mechanism. Phosphorylation of ERM by ROCK2 normally allows ERM to cross‐link actin filaments with the plasma membrane [47]. In contrast, disruption of the endothelial barrier may lead to increased endothelial permeability and promote organ damage in various diseases [48, 49]. 18*β*-Glycyrrhetinic acid, the main active substance of liquorice, has outstanding anti-inflammatory and antioxidant effects, and it inhibited pulmonary vascular remodelling by reducing the expression of RhoA and ROCK2 in pulmonary artery smooth muscle cells [50].

GBEE significantly suppressed NF-*ĸ*B signalling activation by reducing the phosphorylation of I*ĸ*B and the DNA-binding activity of NF-*ĸ*B in the present study. These suppressive effects led to a significant reduction in the expression of the cell adhesion-associated proteins ICAM1, ROCK, and RhoA in EA.hy926 cells. Our previous [7] study showed that GBEE reduced inflammation via reduced NF-*κ*B activation in RAW 264.7 cells. GBEE also reduced metastasis in human colorectal cancer cells via decreased NF-*κ*B activation [51]. NF-*ĸ*B primarily controls the adhesion- and transmigration-related regulatory molecules ICAM1, E-selectin, ROCK, and RhoA at the transcriptional level [52]. The results of the present study indicate that NF-*ĸ*B is an important cellular target of GBEE. GBEE reduction of NF-kB signalling may be one of the important pathways in the suppression of adhesion, cell-cell monolayer permeability, and transmigration.

Notably, the acute oral toxicity study indicated that the methanol extract of *G. bicolor* has a negligible level of toxicity when administered orally and has been regarded as safe in experimental rats [5], and from a hepatotoxic assessment of pyrrolizidine alkaloids in *G. bicolor in vitro*, it is not found toxic effect [53]. Moreover, the leaves of *G. bicolor* exhibit specific colouring of dark green on the top and purple on the bottom. Lu et al. showed that the abundant plant pigments in leaves provide *G. bicolor* with its pigmentation and may have physiological effects [54]. Our previous studies showed that *G. bicolor* was rich in the chlorophyll, flavonoid, and carotenoid families of plant pigments, including gallic acid, *β*-carotene, rutin, anthocyanidin, myricetin, and morin [6, 7]. Bhaskar et al. also showed that flavonoid- and polyphenol-rich materials have the potential to regulate the expression of adhesion molecules [55]. However, identification of the active compounds in GBEE that regulate permeability and cell-cell monolayer integrity requires further study.

## 5. Conclusion

**Figure 6:**
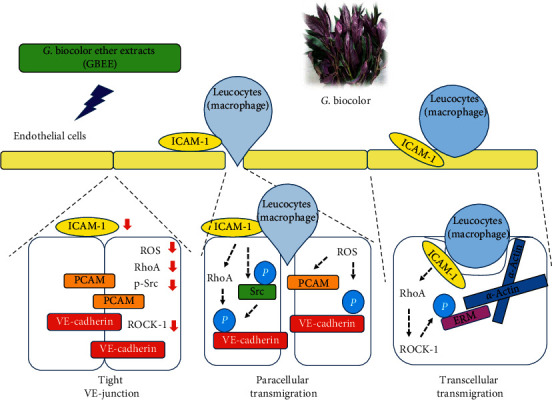
Possible mechanisms of GBEE inhibition of paracellular and transcellular transmigration in EA.hy926 cells.

The present results showed that GBEE had a potential protective effect against atherosclerosis via suppression of leukocyte-endothelium transmigration.  Figure 6 shows that GBEE inhibited adhesion molecules and junction protein expression via inhibition of NF-*κ*B signalling and reduced leukocyte adhesion to endothelial cells ability and endothelial monolayer permeability, which led to the downregulation of adhesion and transmigration-regulated molecular expression. GBEE reduced paracellular and transcellular transmigration to maintain cell-cell monolayer integrity, reduce permeability, and prevent endothelial dysfunction.

## Data Availability

The datasets used and/or analysed during the present study are available from the corresponding author upon reasonable request.
